# Societal views on using risk-based innovations to inform cancer screening and referral policies: findings from three community juries

**DOI:** 10.1186/s12889-025-21996-x

**Published:** 2025-02-27

**Authors:** Rebecca A. Dennison, Reanna J. Clune, Joanna Tung, Stephen D. John, Sowmiya A. Moorthie, Jo Waller, Juliet A. Usher-Smith

**Affiliations:** 1 Department of Public Health and Primary Care, Primary Care Unit, University of Cambridge, Cambridge, CB2 0SR UK; 2 Department of Psychology, University of Cambridge, Cambridge, CB2 3EB UK; 3 Department of History and Philosophy of Science, University of Cambridge, Cambridge, CB2 3RH UK; 4 PHG Foundation, University of Cambridge, Cambridge, CB1 8RN UK; 5 Cancer Research UK, 2 Redman Place, London, E20 1JQ UK; 6 Wolfson Institute of Population Health, Mary University of London, London, Queen E1 4NS UK; 7 School of Cancer and Pharmaceutical Sciences, King’S College London, London, WC2R 2LS UK

**Keywords:** Cancer Screening, Community jury, Health Policy, Personalized Medicine, Risk Factors, Artificial Intelligence, Genetic Risk

## Abstract

**Background:**

Recent advances mean that innovations are emerging that enable better stratification of individuals based on their risk of cancer so that screening or diagnostic investigations can be targeted to those at greatest need. We explored the views of the public, from a societal perspective, of using such risk-based innovations to identify people’s cancer risk and allocating healthcare accordingly.

**Methods:**

We conducted three community juries, each with 7–9 participants. Participants were informed about the topic and potential novel risk-based innovations through a series of presentations from experts and discussions. Polygenic risk scores, geodemographic segmentation, continuous monitoring of biomarkers, minimally invasive tests, artificial intelligence analysis of medical records, and wearable devices were used as examples. The participants then deliberated over the research questions before reporting their verdicts on the acceptability of these novel data-based approaches in principle. Transcripts were analysed using codebook thematic analysis.

**Results:**

All juries found that the proposed risk-based approaches to cancer healthcare were, in general, acceptable. Primarily this was because the approaches would enable use of information in a positive and constructive way. However, there were a number of qualifiers or caveats. In particular, participants highlighted the necessity of using accurate and robust data with a well-evidenced association with cancer risk. They also expressed concerns about unintended consequences such as for insurance, scams or erosion of personal liberty, and the burden to participate in data collection across society. All agreed that opting-out must be straightforward.

**Conclusions:**

Informed members of the public supported the concept of using innovations to estimate cancer risk and inform healthcare. Their priorities for accuracy, data security, participation burden, and personal liberty and choice tended to overlap with those of developers and policymakers. Work to ready these innovations for implementation should continue, with the public’s priorities accounted for in their development and dissemination in order to address any unintended consequences upfront.

**Supplementary Information:**

The online version contains supplementary material available at 10.1186/s12889-025-21996-x.

## Background

Preventing cancer and improving cancer survival are universal priorities within healthcare, and are a crucial aspect of the UK’s 2019 National Health Service (NHS) Long Term Plan [1]. Recent technological and scientific advances mean that new innovations are being developed to support cancer prevention, screening and early diagnosis [2]. These have the potential to enhance estimation of cancer risk for individuals or groups of individuals so that subsequent screening or diagnostic investigations could be better targeted to those at greatest need and most likely to benefit, and away from those more likely to experience harms.

Numerous and varied innovations to predict cancer risk are being developed, and existing approaches are being refined [2]. Some involve a single measurement while others require repeated tests or even constant measurements. These include biosensors to enable continuous monitoring or minimally invasive tests to detect biomarkers in blood, urine, saliva, faecal or other samples [3–5]. Coupled with artificial intelligence and machine learning algorithms, wearable technologies, geodemographic segmentation and electronic health records are also being used to estimate risk [6, 7]. Specific innovations, at various stages of development, include wearable sensors to detect ultraviolet light exposure to estimate risk of skin cancer [8], electrochemical immunosensors to quantify breast cancer biomarkers [9], and deep learning models for analysis of non-imaging electronic health record data to estimate risk of lung or other cancers [10]. Most are tests for specific cancers, but multi-cancer tests are additionally emerging, such as for cell-free circulating DNA in blood [11].

Currently, access to screening and diagnostic tests is largely based on age, sex and/or symptoms. Moving to a healthcare system in which access to these tests would more systematically or formally vary according to the estimated risk of cancer requires not only valid, reliable, accessible, and affordable means of estimating risk but understanding public attitudes towards new risk-based innovations. Although public opinion will form part of the development process for individual, patient-facing innovations, the public’s priorities for risk-based innovations for cancer screening and, in particular, diagnosis are also important but much less studied. This includes both how risk is calculated and how it informs healthcare. Studies of the acceptability of risk-stratified cancer screening have found attitudes to be broadly positive, with members of the public seeing it as a logical approach with a range of potential benefits for individuals as well as the healthcare system [12]. In a recent study, participants were asked to consider preferences for various risk-stratified screening strategies from the perspective of society as a whole. Amid overall acceptability, the public were concerned about screening approaches for people who are unable or unwilling to complete the risk categorisation and the implications for people found to have a low cancer risk, in addition to wanting to be able to understand how their risk would be calculated [13].

Various other studies have shown that engagement in risk assessments is anticipated to be high, including both phenotypic and genetic risk [12–14]. However, concerns have been identified about any use of data that might lead to erosion of choice or delivery in healthcare, concerns that widespread linkage of data might uncover hidden patterns in society and lead to different ways of thinking about healthcare, and concerns about the introduction of tiered societal systems with stratification based on genetics [15].

## Methods

### Aim

This study aimed to explore the views of the public, taking a society-level perspective, of using a variety of innovations to identify people’s risk of cancer and using this to prioritise them for screening or diagnostic tests. It was conducted with a view to informing the planning, development and implementation of future risk-based innovations.

### Study design

Community juries enable participants to be informed about the issue in question, consider each other’s views and think beyond their own perspective through facilitated and unfacilitated discussions before reaching a group agreement on the questions posed by the researchers [16].

We conducted three community juries in March and May 2023 and reported these according to the CJCheck Framework [17]. Two juries comprising of two sessions held on consecutive days were conducted online using Zoom video conferencing. One in-person jury was conducted in a University of Cambridge departmental building, lasting one full day. Each session lasted 3–4 h.

### Research team

Seven researchers with experience in the topic and methods led this study, including public health researchers, health psychologists and academic clinicians (a general practitioner/associate professor of general practice and academic clinical fellow). Three primarily contributed to the planning, facilitation and analysis of the juries, and others delivered the presentations on their area of expertise.

Patient and public involvement (PPI) members contributed to the formation of the research question and design of the study from the funding application stage. They were then involved in protocol development and design of participant-facing aspects of the research, such as information sheets and presentations used within the juries, plus interpretation and creation of lay summaries of the findings. Three PPI representatives were known to the research team through working together on related projects, and a fourth representative was recruited via a local institutional PPI panel. Collectively, the PPI representatives represented different demographic groups including by age, sex, ethnicity and personal history of cancer.

### Participants and recruitment

A market research company purposefully recruited jury participants by age, sex, socioeconomic background, and screening history to represent a range of demographics within the study. As those likely to encounter risk-based innovations, participants between 21 and 79 years were eligible. Individuals with a personal history of cancer, medical expertise or who had participated in previous community juries conducted by the research team were not eligible to take part. Those participating in the online juries were resident across the UK, whilst only people able to travel to Cambridge could attend the in-person jury. The market research company supported administration and communication with the participants (including gaining informed consent, sharing relevant organisational information, and reimbursing participants at their recommended rate).

### Jury procedure

RD and RC facilitated the juries. After an introduction to the study, session 1 involved watching four pre-recorded presentations (15–20 min duration) that detailed the key clinical, ethical and regulatory concepts for future risk-based innovations (Table 1). Each was produced by one of the authors, who subsequently joined online for live questions and answers. The fourth presentation included examples of novel innovations that had been identified and prioritised using a survey completed by 20 international academics in the field: polygenic risk scores, geodemographic segmentation, continuous monitoring of biomarkers, minimally invasive tests, artificial intelligence analysis of medical records, and wearable devices (summarised in Supplementary Table 1, Additional File 1). The researchers emphasised to the participants that the innovations would be fully developed, validated, feasible and that the appropriate infrastructure for each innovation would be in place prior to implementation to enable discussions to focus on acceptability rather than other implementation challenges.

**Table 1:** Overview of expert presentations used to inform jury participants

1. Introduction to concepts	• What is prevention, screening and early detection • Current pathways for cancer screening and referral • Current approaches to decision-making
2. Risk prediction	• What is risk prediction • How risk-based innovations could impact practice • Methods of data collection • Data storage • Data access • The importance of public support
3. Ethical considerations	• Resource-limited setting • Principles for considering the ethical issues (beneficence, non-maleficence, justice, and respect for autonomy)
4. Potential impact of risk-based innovations	• Timescales for implementation • Six examples of risk-based innovations: o PRS o Geodemographic segmentation o Continuous monitoring of biomarkers o Minimally invasive tests o AI analysis of medical records o Wearable devices • Individual impact at different points on the pathway • Impact on healthcare systems • Outstanding research questions

*AI* Artificial intelligence, *PRS* Polygenic risk scores

Session 2 began with a focus group discussion designed to encourage participants to consider and share their views as a group (facilitated discussion 1; see topic guide developed for this study in Additional File 2). RC and RD remained impartial by withholding their own views and expertise, and promoting a range of perspectives. After making sure participants understood the questions, the researchers left the participants to seek an agreement on the research questions shown in Table 2 (unfacilitated deliberation). Once the participants were ready, they shared their verdicts with JUS (feedback session). Finally, the participants considered barriers and facilitators to acceptability in a final focus group informed by the theoretical framework of acceptability (TFA) [18] (facilitated discussion 2).

**Table 2:** Research questions for deliberation and verdict

1. Do you think it is acceptable, in general, to use data from a range of sources to assess cancer risk and use that risk to determine access to healthcare?
2. Does it make a difference whether people have symptoms or not?
3. Does it make a difference what data are used?
4. Do you think people need to have the option to opt out of the use of data being used in this way? If so, how would you handle those individuals?
Please be prepared to explain your answers

The questions were explained to the participants before the researchers left the deliberation to ensure understanding

### Data collection and analysis

All aspects of the jury apart from the presentations were videorecorded and transcribed verbatim. The transcripts from session 2 were analysed using codebook thematic analysis [19] in order to thoroughly explore the participants’ verdicts and support for or rejection of the innovations. A high-level coding structure was outlined based on the research questions and topic guide; the inductive coding frame was then developed to account for participants’ discussions. One or two researchers coded each transcript using NVivo 12 software (Lumivero, Colorado, US). Through discussion with the research team, themes were developed in order to explore the reasons behind the juries’ answer to each research question. For question 3, we focussed on identifying unifying principles that influenced acceptability rather than the specific merits or flaws of each example of innovation.

Participants also completed supplementary questionnaires before and after the juries (Additional File 2). These used validated or published measures as indicated; if validated measures were not available, the research team drafted questions and checked them with the PPI representatives for understandability. Before the juries only, participants were asked to provide:

- Categorised demographic information: age group, sex, gender identity, simplified ethnicity and educational attainment;
- Health and screening history: general self-perceived health status, family history of cancer, and whether they had ever attended bowel, breast or cervical cancer screening or abdominal aortic aneurysm screening;
- Thoughts about cancer and screening: six items about general cancer beliefs based on the awareness and beliefs about cancer (ABC) measure [20], self-perceived likelihood of developing cancer, and three items from the attitudes towards general health check-ups subscale of the breast cancer screening beliefs questionnaire [21];
- Online privacy attitudes and behaviours: four items selected from the privacy attitudes and technical protection subscales from the internet privacy attitudes and behaviour multi-dimensional scale [22].

We also sought participants’ attitudes towards risk-based innovations in order to identify any individual changes before and after the juries. This used a simplified version of the four questions posed in the juries with responses given on a 5-point Likert scale. Finally, they gave their experience of participating in the community jury at the end of the study using a questionnaire (shared by personal communication) developed to evaluate the goals of community juries [23], which we have also used previously [13, 24]. The questionnaires were analysed using descriptive statistics in Microsoft Excel.

## Results

### Participant characteristics

Twenty-four participants took part in the three juries: 17 online and nine in-person. Individuals with a variety of demographics made up each jury (Table 3), with the exception of jury 2 where all but one participant was of white ethnicity and jury 3 where two female participants withdrew immediately before the start due to unforeseen factors resulting in a larger proportion of males. There was an even distribution of participants with lower and higher occupational social grades, and 46% had a degree.

**Table 3:** Participant demographics

	**Jury 1**	**Jury 2**	**Jury 3**	**Total (%)**
	**Online**	**In-person**	**Online**	
**Total *N***	8	9	7	24 (100.0)
**Age (years)**				
21–39	4	3	1	8 (33.3)
40–49	1	3	1	5 (20.8)
50–59	1	1	2	4 (16.7)
60–79	2	2	3	7 (29.2)
**Sex**^**a**^				
Female	4	4	2	10 (41.7)
Male	4	5	5	14 (58.3)
**Simplified ethnicity**				
Asian	1	1	0	2 (8.3)
Black	1	0	1	2 (8.3)
Mixed or multiple ethnic group	1	0	1	2 (8.3)
White	5	8	5	18 (75.0)
**Occupational social grade**				
Lower social grade	3	5	4	12 (50.0)
Higher social grade	5	4	3	12 (50.0)
**Education level**				
Not completed A levels, further education or equivalent	3	3	1	7 (29.2)
Competed A levels, further education or equivalent	3	1	2	6 (25.0)
Completed a bachelor’s or postgraduate degree	2	5	4	11 (45.8)
**Self-perceived health status**				
Fair	2	2	3	7 (29.2)
Good	2	4	1	7 (29.2)
Very good or excellent	4	3	3	10 (41.7)

^a^For all participants, gender identity corresponded to sex as registered at birth

Additional participant characteristics, including their thoughts about cancer and screening, are shown in Supplementary Table 2, Additional File 1. Ten participants had a family history of cancer. Only one participant would not want to find out if they had cancer. The participants provided positive feedback regarding their experiences of participating in the juries; see Supplementary Fig. 1, Additional File 1.

The juries’ verdicts on the research questions are explained below and supported by quotes from throughout the discussions.

### Question 1: Acceptability of the principle

All participants perceived estimating risk of cancer using novel innovations and using the outcome to inform cancer screening and diagnosis policies favourably, with all three juries concluding that the principle was acceptable. However, all included a caveat within their verdicts, anticipating the potential for both positive and negative impacts of introducing risk-based innovations.

“*We were happy with the in general use of data. It's proven to make a difference but perhaps, not become too over reliant on it because it's one factor, basically*.” [P1-1 (jury-participant number), feedback session]

“*We all agreed that, yes, it is absolutely fine to do that. But saying that, as long as […] we’re not excluding people who don’t necessarily fulfil the criteria but have a genuine cause for concern, to have a screening or a test*.” [P2-17, feedback session]

“*So as a group we do believe it is acceptable to use data and use modern techniques, provided the sources are ethical and medical and the data is accurate*.” [P3-22, feedback session]

These views were based on the juries’ discussions of the possible implications on six key domains (detailed in Table 4). They considered that the pros of such a change would outweigh the cons in each case. For example, they did not want healthcare professionals’ clinical expertise, intuition and relationship with their patients to be replaced by risk assessments – hence jury 1’s proposition that the risk assessment is only one of the considerations when making referral decisions or screening policies – but supported the implementation of risk-based innovations overall because of the greater potential benefit of enabling healthcare to become more efficient and prioritise those in greatest need.

**Table 4:** Anticipated impacts of using novel innovations to identify people’s risk of cancer and using this to prioritise them for screening or diagnostic tests

	**Con/disadvantage**		**Pro/advantage**	
**Domain of impact**	**Summary**	**Illustrative quotation**	**Summary**	**Illustrative quotation**
Healthcare system	Conducting a risk assessment could result in more work overall, and innovations could reduce human interaction with healthcare professionals	“The problem is people will be alarmed because they’re losing that personal interaction and maybe because people’s fear of automation…” [P3-25, FD1]	It has the potential to enable healthcare services to become more efficient and proactive, meaning care could be prioritised to those who need it most	“…they are now becoming proactive rather than reactive, because the NHS is very reactive at the moment.” [P3-27, FD2]
Cancer outcomes	Delays in diagnoses and treatment could result from incorrect classification of risk, or in people with a low risk who still develop cancer	“Let’s say someone who’s not in that risk factor but does turn out to have cancer but was excluded from that process. It could cause a social dilemma… the chances are it’ll end up on the news or the Daily Mail [newspaper] and BBC News. That can turn from being a social dilemma to being politically unpopular. That can ruin the reputation of the NHS if it’s quite widespread.” [P1-5, FD1]	This has the potential to facilitate earlier detection and improved outcomes	“Obviously, everything comes with pros and cons, and if they use the data to narrow the field, you are going to potentially miss more, but you’re potentially going to save more as well.” [P2-18, FD1]
Behaviour and decision-making	Knowing more about your cancer risk has the potential to impact life decisions significantly and negatively (such as choosing not to save for retirement if you have a high cancer risk)	“What’s the point in paying off my mortgage because I’m going to be dead before…” [P1-6, FD1]	Knowing more about your cancer risk could motivate positive action (such as making healthy lifestyle choices)	“Obviously, you can’t change genetics and you can’t change your age, and you can’t change, you know, that sort of thing, but you can change some parts of your lifestyle.” [P2-18, FD2]
Psychological	Discovering your risk or taking part in a risk assessment could result in anxiety (for example, because of the awareness of every aspect of life impacting cancer risk and/or constant monitoring)	“I was quite happy, toddling along as I was and now, “Oh my. What might happen next week? … At the same time, I’d know it was great, but I’d also worry.” [P1-1, UD]	Discovering your risk could result in reassurance (for example, because you are found to have a low risk or, even if you have a high risk, you would be monitored so cancer could be treated early)	“The anxiety is that something’s possibly been identified. I’d cooperate and it would either confirm or dispel… it’s not just accept, I would be reassured. And actually, I suppose I’d feel valued within the system.” [P3-25, FD1]
Technological advances	Concerns that implementing risk-based innovation is changing the system just for the sake of it	“It’s almost like one’s going to replace what is working at the moment, currently working, or appears to be working, shall I say, in my opinion, rather than something that’s going to be – change for change’s sake, shall we say.” [P2-14, FD1]	This is an opportunity to utilise technological advances and additional data, and adding another ‘layer’ in policies is more logical than basing them on age and sex	“The medical industry is evolving, they’re using information in a positive and a constructive way.” [P3-25, FD1]
Fairness and ethics	In some ways, it is unfair to prioritise the health of people who choose to have unhealthy/risky lifestyles (although universal healthcare is inherent to society’s wider values)	“I think people can make their own choices and I’m all for being fair and stuff like that. I do feel those more at risk should take priority. Like if somebody smoked all their life and drank all their life and then, gets priority over somebody else that hasn’t, I do think that’s unfair, and it would cause resentment.” [P1-7, FD1]	Differential screening/testing is acceptable as long as it is justified, policies change in light of new data, and people are ‘taken seriously’ if they have symptoms	“I think I wouldn’t have a problem with it if I knew that they were at higher risk. Yes. Like my dad gets screened for bowel cancer but I don’t because I’m not that age yet.” [P1-6, FD1]

Participant identification in the format P(participant number)-(jury number)

*BBC* British Broadcasting Corporation, *FD1/2* Facilitated discussion 1/2, *NHS* UK National Health Service, *UD* Unfacilitated deliberation

Notably, they felt that the introduction of risk-based innovations would be a large or radical change to healthcare policy. They anticipated varying levels of public support because “*It’s a very, very big undertaking to shift people’s attitudes*” [P1-1, facilitated discussion 2], and some opposition from the media.

### Question 2: Differences in acceptability for people with and without symptoms

The stated conclusions of all juries were that using risk-based innovations was equally acceptable to determine access to screening tests as to diagnostic tests.

*“So generally we said no, it doesn’t make a difference, symptoms or not, for the risk-based approach. Everybody was on the same page with that one.”* [P3-22, feedback session]

However, analysis of their group discussions and follow-up questioning in the feedback sessions revealed several complex viewpoints that are potentially in tension with the verdict, as well as suggestion of some misunderstanding by some participants. In these discussions, the principle of risk stratification appeared to some to be more logical when applied to screening than when applied to referral to investigate symptoms. It was clear that it is not valuable to screen people with a low risk of cancer, whereas “*if you’ve got symptoms you should get the same test*” [P1-8, unfacilitated deliberation] even with a low cancer risk. On the other hand, the benefits of apparently healthy people completing a ‘medical’, invasive or intrusive test for the risk assessment were less obvious to some participants, from the perspective of both the individual undergoing testing and the healthcare service committing scarce resources.

*“When all the talking stops and you’ve got a really limited resource, with a massive backlog waiting list, if you were them, surely you would go for symptomatic rather than non-symptomatic. When it actually comes to the crunch, you can test everyone but symptomatic must lead to better outcomes, early interventions, people actually need something rather than maybe need something.”* [P1-8, unfacilitated deliberation]

*“We would be conscious if it was a thing that we had to wear it all the time, if we weren’t showing symptoms. As opposed to… we just presumed we’d wear it if we were showing symptoms, to further the investigation.”* [P1-7, feedback session]

This suggests that acceptability is nuanced and depends on situation-specific factors including the degree of medicalisation and intrusiveness of the risk assessment method, vagueness or seriousness of symptoms, and availability of resources.

### Question 3: Principles of acceptable innovations

The participants were generally positive about all six examples of risk-based innovations presented to them. Their priorities for acceptable risk assessments were accurate risk classification, data security, maintaining personal liberty, and minimising the burden or effort required to participate. If they could be reassured that these conditions were met, they largely would be happy for them to be implemented.

#### Accurate classification of risk

The participants required confidence that risk of cancer would be estimated with sufficient accuracy and not misclassified. Misclassification as high risk could lead to avoidable anxiety and inappropriate testing. Misclassification as low risk could delay diagnosis and treatment to the detriment of the individual’s health as well as the reputation of the policy. Many therefore felt that risk should only be used alongside clinical expertise so that decisions would not be based on the risk estimate alone, especially if someone had a low estimated risk of cancer and in the context of referral to investigate symptoms.

In order to avoid misclassification, the association between the data collected and cancer risk must be robust. For example, it was intuitive that using geodemographic segmentation data based on postcode would be invalid due to large differences in environmental risk factors such as pollution levels within postcodes. Similarly, whilst some felt it was “*okay to be a little bit grey*” [P2-17, unfacilitated deliberation], others viewed the ‘black box’ nature of AI algorithms with caution because it meant risk assessments could not be verified, adjusted manually or fully explained.

*“…but it could be that there is an unconscious bias in the institution that means that it’s not really looking at everything in the best interests of everybody, it’s not representative of the entire population.”* [P3-23, unfacilitated deliberation]

Furthermore, it was important that any technology is suitably accurate to collect and record data in order for the risk assessment to be correct. Several participants expressed concerns over the accuracy of wearable devices to record physical parameters (comparable to variability when measuring heartrate) or that they could be intentionally manipulated. Additionally, data could be old or missing, such as housing data that has the potential to change frequently and not be kept up to date on medical records. The ability to easily access, check and update risk data was important.

#### Data security

The participants also stressed the need for the establishment of appropriate safeguards, namely data protection laws rather than a code of conduct. They anticipated a range of unexpected and unintended consequences, and wanted the safeguards to include data on both the risk factors (particularly genetic data) and risk classification.

Specifically, all juries raised concerns about data being shared with, sold to or even hacked by external agencies. They tended to trust the NHS to hold personal data but were suspicious about third-party or private companies, including if the NHS outsourced tasks to them. Moreover, the idea that “*these tests [could] penalise you from something else […] for years to come*” [P1-7, facilitated deliberation 1] was raised across all three juries. For example, insurance companies could charge higher rates for people with higher risk of illness (which could further compound the challenges faced by many people in these positions). Importantly, this would impact everyone, even if they chose not to take part in the risk assessment or not to disclose the result, as the absence of risk information could be used against them.

*“…we’re all happy to participate but the minute that that cooperation is used against us then the cooperation is withdrawn [… imagine] a situation where just through prudence you had a test done because of what was going on within your family and the test that you had was actually possibly used against you.”* [P3-25, unfacilitated deliberation]

*“…those who are going to be ill are going to bear the burden of it, because they won’t get insurance, they won’t get all the other things…”* [P2-14, facilitated deliberation 1]

#### Personal liberty

“*The onslaught of Big Brother*” [P3-25, unfacilitated deliberation] was also considered important by many participants across the juries, meaning that the approach had the potential to be too intrusive on personal freedom or liberty. They compared it to other contexts such as targeted online adverts and virtual assistant technologies ‘listening’ to their conversations, although they had little control and gained minimal benefit in those. This was particularly relevant to risk assessments based on lifestyle such as wearable devices because they felt that people should be free to make decisions about their lifestyle and that risk assessments could result in the individual being blamed for their higher cancer risk. Conversely, other participants did not share these concerns. Some felt that they did not have anything to hide and others realised that they could benefit personally through more appropriate cancer screening and investigations, therefore it was worth their while.

*“I guess it does make me slightly uncomfortable but then, I also think, well, these commercial companies are collecting all of this information anyway. The NHS would put that information to much better use than they would by trying to sell me stuff I don’t want. I feel slightly uncomfortable about it but I can, kind of, see the benefits as well.”* [P1-6, feedback session]

*“I’d fully cooperate if it’s in my wants and best interest to give as much information […]”* [P3-25, facilitated deliberation 1]

#### Burden of participation

Lastly, participating in a risk assessment was not considered to be asking too much of members of the public, particularly if the option to opt-out was available (see question 4). The tests were likened to vaccinations and existing cancer screening that many people already engage in.

The participants preferred risk assessments that were easier and quicker to complete. This included using data that are already available such as running an AI algorithm on medical records, or the “*quick wins*” [P1-1, feedback session] of risk assessments based on blood or minimally invasive tests. Some participants were comfortable wearing a patch to continuously monitor biomarkers “*as long as it’s not too uncomfortable*” [P1-6, feedback session].

However, the participants did acknowledge that some people would find it easier to complete the tests or be more willing to engage in them than others, and that those with most difficulty or reluctance could be people who are already disadvantaged in society. It was therefore felt that tests should be made as easy as possible to access by providing them in supermarkets, places of work, pharmacies, etc.

*“If you’re a mum, juggling a full-time job and caring for your kids, trying to find the time to go to your GP and have the test might be tricky. But if you want to look after your health, you prioritise things, don’t you?”* [P1-6, facilitated deliberation 1]

### Question 4: Views on opting in versus opting out

Finally, the participants predicted that some members of the public would want to take part and discover their cancer risk whereas others would not. It was therefore essential that people have the choice of whether to take part in the risk assessments. All juries took the viewpoint that these policies should be implemented automatically and people should opt-out if they did not wish to take part. Jury 3 was concerned that people in certain demographic groups might miss out on the better approach otherwise.

*“It wouldn’t bother me at all if everybody in the whole world’s seen my data but as long as I’ve got the option to choose. It’s up to me to say, ‘Yeah, you can have it’ or ‘No, you can’t’…”* [P3-29, facilitated deliberation 1]

*“I think everyone should be in and they opt out if they want to. I think if you do it the other way round there might be people who aren’t aware of what’s going on and will be opted out without being able to change it, particularly perhaps the elderly and that sort of thing, who are not sort of so in touch with what’s going on or tech-savvy.”* [P3-27, unfacilitated deliberation]

People should be able to make informed decisions by having sufficient information about the policy and understanding the consequences of opting out. Furthermore, it should be straightforward for individuals to implement their preferences according to type of data. Respecting individual choices in this way would make it fair.

However, opinions on how to screen or refer individuals if they did not have a risk assessment varied. Many participants felt that it was important for people who opt out not to be disadvantaged, particularly that they would not “*be treated any different to anybody else at a later date if they present with symptoms; they shouldn’t be penalised*” [P1-8, unfacilitated deliberation]. However, they were not clear what it would mean in practice for them to “*just remain on the standard procedure and follow the usual route*” [P3-22, feedback session]. Participants in juries 1 and 3 felt that those who opted out should be managed as average or low risk, meaning that they should accept less frequent screening or initial referrals for less intensive tests.

*“There have to be consequences to opting out… In a caring, compassionate society, they’re still going to get looked after but if it’s at a slightly lower level of intensity, if that’s the word, then so be it.”* [P1-1, feedback session]

### Individual views

**Fig. 1:**
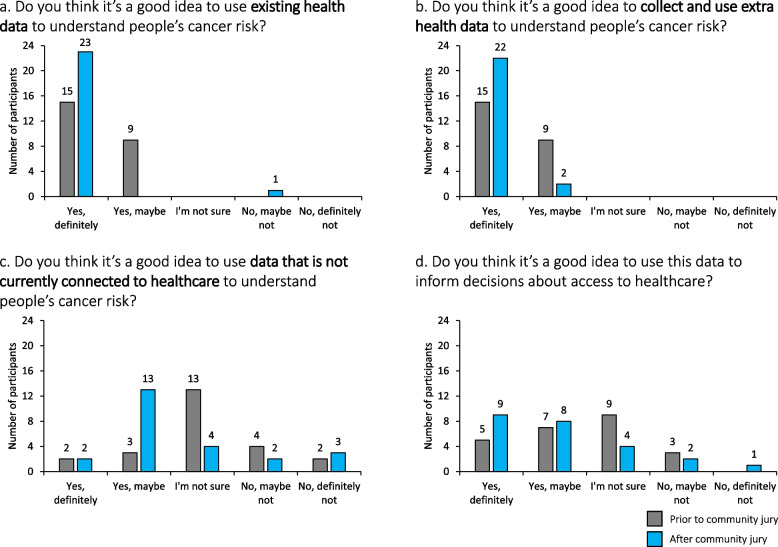
Participants’ individual beliefs about using data to estimate risk of cancer before and after the community juries. *N* = *24 participants*

When asked for their individual views in questionnaires before and after the jury discussions (Fig. 1), participants tended to be more positive about using personal data in cancer healthcare after taking part in the juries than before. All participants except one thought it was a good idea to utilise existing or newly collected health data; eight participants had increased their support for these strategies after taking part. There were more differences in views towards using data that are not currently connected to healthcare, with 14 participants being more sure about their use after the juries but also five participants being less sure. They were also less sure about using the risk assessment to inform decisions about cancer healthcare overall, but 17 (70.8%) supported the idea after the juries compared to 12 (50.0%) before the juries.

## Discussion

### Key findings

Our findings suggest that the public are receptive to the concept of risk-stratified cancer screening and diagnosis in which risk is predicted using innovative methods. Many may even be eager for such a change, anticipating that new technologies could improve the health of individuals and society, and help the health service by increasing efficiency.

**Fig. 2:**
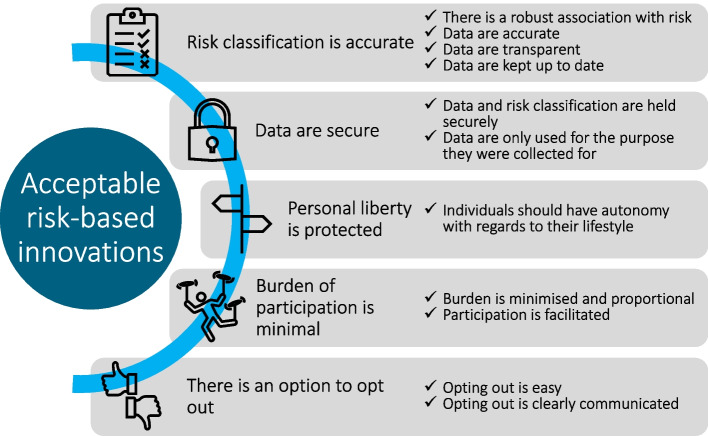
Summary of participants’ requirements for acceptable risk-based innovations

In order to be acceptable, the benefits (such as on personal outcomes) of any innovation should outweigh the costs (including anxiety, inconvenience and loss of freedom). Participants expected the gains to be greater if they had symptoms, so were willing to ‘pay’ more and accept greater costs in this context than if they are asymptomatic. Based on these findings, requirements for risk-based innovations to be acceptable to the public are shown in Fig. 2. Innovations and risk-based policies should be developed to prioritise an accurate risk classification. They should take into consideration the actual and perceived reliability of both the data going in and coming out (i.e. the risk classification), although participants did not discuss what degree of misclassification, if any, they would accept. The effort required to participate should not be greater than necessary and participation should be made as straightforward as possible. Policies should ensure that individual autonomy, with regards to whether to take part in the risk assessment and to make lifestyle choices, is protected and they should not miss out on healthcare from not taking part. Lastly, data on cancer risk factors and risk classification should be protected by law and through secure storage and transfer so that unintended consequences do not occur.

Additionally, it is likely that the public will need to be able to understand how these requirements are enacted. For example, the participants were willing to be somewhat inconvenienced by a test as long as they could understand why that risk assessment was relevant and be confident that it would benefit them in terms of a better cancer screening policy or appropriate referral. Consequently, mass and individual-level communication will be essential.

### Comparison to existing literature

The support for use of risk-based innovations within screening is consistent with public views expressed in other studies focused on risk-stratified screening that is based on genetics or ‘typical’ risk factors such as age, sex, family history and lifestyle characteristics. Across those studies, risk stratification was considered to be logical and associated with wider benefits than the universal screening approach that is currently used [12]. The public have also previously shown positive views towards complex risk prediction strategies, including using genetic risk [14, 25]. Our study highlights that many of these priorities and concerns also apply in the context of implementing novel innovations within referral decisions for people with symptoms. For example, that the public value early diagnosis and discovering their risk of cancer, yet it should remain optional [26, 27]. Given the known eagerness for cancer testing to investigate symptoms, even if the individual has a very low risk of cancer [28], the public in this study may have mistakenly equated completing a test for the purpose of a risk assessment with having an investigation for symptoms. Additionally, whilst concerns about the insurance implications for genetic risk assessments have been observed before [29], it is notable that participants in our study were concerned about such implications relating to all forms of risk assessment and in contexts wider than health or life insurance.

The priorities of the public for the use of novel innovations have important overlap with those of researchers, developers and policy makers. These shared priorities suggest that it is insufficient just to implement risk-based innovations or even communicate any changes with professionals, but that this information must be accessible to the public. For instance, the authors of a recent review identified a need to address issues around data security, privacy and accuracy when considering the implementation of wearable medical devices; the authors also highlighted lack of industry standards and regulations, equipment safety, battery life and user-friendliness, which are similar to topics that our participants discussed [30]. Additionally, the public’s requirements for risk assessments pick up on aspects of the refined consolidated screening principles: the accuracy requirement is comparable to the principles of screening test – which equates to risk assessment – performance and interpretation of results (principles 4 and 5) and overall benefits and harms (principle 10) [31]. Moreover, a recent UK National Screening Committee modelling event commented on the importance of accurate data in risk stratification, highlighting that ‘bad data equals a bad model’ [32]. The principles of medical ethics also pick up on similar points, adding the importance of respecting individual autonomy [33, 34].

### Strengths and limitations

The community jury method used in this study is particularly suited to topics such as this where there is the need to balance a range of advantages and disadvantages across a range of dimensions. The inclusion of expert presentations increased the likelihood that the opinions expressed were thought-through and the relatively large amount of time given to participants to consider their own as well as others’ views through multiple discussions enabled us to explore views from the perspective of wider society. Despite this, some misunderstanding was evident in the discussions. For example, we needed to assure jury 2 that investigations for someone presenting with a breast lump would not be delayed if they had a low risk of cancer because this is a specific symptom which, in itself, meets the risk threshold for referral. While expert presentations have the potential to influence the participants, we sought to present information objectively from multiple perspectives and to highlight uncertainty. The participants also had the opportunity to challenge or question the experts. Furthermore, the facilitators came from a range of backgrounds, took neutral perspectives and encouraged participants to express their opinions.

The hypothetical nature of the research questions may have increased the difficulty of the exercise. We explained examples of innovations broadly without providing details such as exactly what would be involved/measured or accuracy, which was in part due to the stage of development of these technologies. We could only confirm that such information would be known prior to implementation. Nonetheless, participants were able to draw on real experiences (e.g., with smartwatches), making each innovation more tangible.

A range of demographic characteristics were included in the sample, such as across the spectrum of education levels. However, it is probable that the perspectives of those who did not express interest in taking part would be different to those who did. Furthermore, most of jury 2 were of white ethnicity and most of jury 3 were male, which may have made the participants with under-represented characteristics feel less able to share their views. While it was not possible to balance the sample on the day, we mitigated this as best we could; for example, by explaining to jury 3 that female participants had withdrawn last-minute.

## Conclusions

Informed members of the public supported the principle of using novel innovations to estimate cancer risk and using the result within risk-based healthcare. This support was, however, conditional and we identified requirements for acceptable polices and risk assessments across five areas: accuracy of the risk assessment, data security, personal liberty, participation burden and choice to opt-out. These priorities should be taken into account when developing novel technologies, planning implementation of risk-based innovations, and communication about both the innovation itself and any associated policies, such as those relating to data security, to the public.

## Supplementary Information

Additional file 1: Supplementary tables and figures. Supplementary Table 1. Summary of the risk-based innovations used as examples in this study. Supplementary Table 2. Additional participant characteristics. Supplementary Figure 1. Participants evaluation of the community juries

Additional file 2: Questionnaires and topic guides. Pre-jury questionnaire. Post-jury questionnaire. Facilitated discussion topic guide outline

## Data Availability

The dataset supporting the conclusions of this article (pseudo-anonymized transcripts) and study materials (protocol, participant information sheet, consent form, participant information pack, facilitated discussion topic guide, and questionnaires) are available via the University of Cambridge Data Repository, 10.17863/CAM.107283 . Formal requests for access will be considered via a data-sharing agreement that indicates the criteria for data access and conditions for research use and will incorporate privacy and confidentiality standards to ensure data security.

## Abbreviations

AI: Artificial intelligence
NHS: National Health Service [of the United Kingdom]
PPI: Patient and public involvement [panel]
PRS: Polygenic risk score
TFA: Theoretical framework of acceptability
