# Supplementary material for: Societal views on using risk-based innovations to inform cancer screening and referral policies: findings from three community juries
Source: BMC Public Health. 2025 Feb 27;25:801. doi: 10.1186/s12889-025-21996-x (PMC11869612; doi:10.1186/s12889-025-21996-x)
Supplement: Supplementary file 1 — Additional file 1: Supplementary tables and figures. Supplementary Table 1. Summary of the risk-based innovations used as examples in this study. Supplementary Table 2. Additional participant characteristics. Supplementary Figure 1. Participants evaluation of the community juries [file 12889_2025_21996_MOESM1_ESM.pdf]

Societal views on using risk-based innovations to inform cancer screening and referral policies: Findings from three community juries

Dennison RA, Clune RJ, Waller J, Tung J, John SD, Moorthie SA, Usher-Smith JA

Additional file 1

Supplementary Table 1. Summary of the risk-based innovations used as examples in this study.

	How can it be used to assess cancer risk?	How does someone take part?
Polygenic risk scores	A polygenic risk score accounts for the many variations in someone's genes. Some of these increase the risk of certain cancers while others protect against them.	By providing a blood or saliva sample for analysis.
Geodemographic segmentation	By considering geodemographic data, generalisations about cancer risk can be made (e.g. population density or pollution levels).	By providing their postcode and additional data such as about their housing or socioeconomic situation.
Minimally invasive tests	Biomarkers can be used to understand processes occurring in the body. They may be produced by the cancer cells themselves, or other cells in response to the cancer. A minimally invasive test would provide a snapshot of someone's biomarkers.	By providing a blood, saliva, urine, stool or sweat sample for analysis using a non-invasive or minimally invasive test.
Continuous monitoring of biomarkers	Unlike the snapshot generated by a minimally invasive test, continuous monitoring would reveal changes in someone's biomarkers over a period of time.	By wearing a patch or other sensor to constantly measure certain biomarkers.
Artificial intelligence (AI) analysis of medical records	An AI algorithm could be applied to someone's medical record (e.g. age, sex, smoking status, past medical history, medications, etc.) and generate a cancer risk score.	By consenting to their health records being analysed.
Wearable devices	A wearable device could continuously track, record, and monitor physical health parameters. For example, sleep patterns or temperature of the skin of the breast.	By wearing the device and sharing the data with clinicians.

Supplementary Table 2. Additional participant characteristics.

	Jury 1 Online	Jury 2 In-person	Jury 3 Online	Total (%)
Total N	8	9	7	24 (100.0)
Familiarity with cancer and screening				
Family history of cancer				
Yes	3	4	3	10 (41.7)
No, don't know or prefer not to say	5	5	4	14 (58.3)
History of cancer in a close friend				
Yes	1	4	6	11 (45.8)
No, don't know or prefer not to say	7	5	1	13 (54.2)
Have completed screening for:				
Abdominal aortic aneurysm	1	2	1	4 (16.7)
Bowel cancer	2	2	3	7 (29.2)
Breast cancer	1	0	1	2 (8.3)
Cervical cancer	4	3	2	9 (37.5)
Thoughts and beliefs about cancer				
"These days, many people with cancer can expect to continue with normal activities and responsibilities"				
Strongly agree or agree	5	5	4	14 (58.3)
Neither disagree nor agree	2	3	1	6 (25.0)
Strongly disagree or disagree	1	1	2	4 (16.7)
"Most cancer treatment is worse than the cancer itself"				
Strongly agree or agree	2	6	4	12 (50.0)
Neither disagree nor agree	4	2	1	7 (29.2)
Strongly disagree or disagree	2	1	2	5 (20.8)
"I would not want to know if I had cancer"				
Strongly agree or agree	1	0	0	1 (4.2)
Neither disagree nor agree	1	1	0	2 (8.3)
Strongly disagree or disagree	6	8	7	21 (87.5)
"Cancer can often be cured"				
Strongly agree or agree	5	7	4	16 (66.7)
Neither disagree nor agree	1	1	0	2 (8.3)
Strongly disagree or disagree	2	1	3	6 (25.0)
"Going to the doctor as quickly as possible after noticing a symptom of cancer could increase the chances of surviving"				
Strongly agree or agree	8	8	7	23 (95.8)
Neither disagree nor agree	0	1	0	1 (4.2)
Strongly disagree or disagree	0	0	0	0 (0.0)
"A diagnosis of cancer is a death sentence"				
Strongly agree or agree	3	2	2	7 (29.2)
Neither disagree nor agree	2	5	0	7 (29.2)
Strongly disagree or disagree	3	2	5	10 (41.7)

Compared with other people the same age and sex as you, what do you think your chances of getting cancer in the next 10 years are?				
Below or much below average	1	0	2	3 (12.5)
Same as always	3	9	2	14 (58.3)
Above or much above average	4	0	3	7 (29.2)
Thoughts and beliefs about screening				
"If I feel well, it is not necessary to have cancer screening"				
Strongly agree or agree	1	0	1	2 (8.3)
Neither disagree nor agree	1	1	0	2 (8.3)
Strongly disagree or disagree	6	8	6	20 (83.3)
"If I follow a healthy lifestyle such as a balanced diet and regular exercise, I don't feel it is necessary to have regular screening"				
Strongly agree or agree	2	0	1	3 (12.5)
Neither disagree nor agree	0	1	0	1 (4.2)
Strongly disagree or disagree	6	8	6	20 (83.3)
"I see a doctor or have cancer screening only when I have a health problem"				
Strongly agree or agree	3	3	3	9 (37.5)
Neither disagree nor agree	1	4	2	7 (29.2)
Strongly disagree or disagree	4	2	2	8 (33.3)
Attitudes towards online privacy				
In general, how concerned are you about your privacy while you are using the internet?				
Extremely or very	2	2	0	4 (16.7)
Moderately	3	4	4	11 (45.8)
Slightly or not at all	3	3	3	9 (37.5)
Are you concerned that you are asked for too much personal information when you register or make purchases online?				
Extremely or very	2	2	2	6 (25.0)
Moderately	3	5	1	9 (37.5)
Slightly or not at all	3	2	4	9 (37.5)
Are you concerned who might access your medical records electronically?				
Extremely or very	1	2	2	5 (20.8)
Moderately	1	1	2	4 (16.7)
Slightly or not at all	6	6	3	15 (62.5)
Do you watch for ways to control what people send you online?				
Rarely or sometimes	3	5	3	11 (45.8)
Often	3	4	3	10 (41.7)
Always	2	0	1	3 (12.5)

Supplementary Figure 1. Participants evaluation of the community juries.

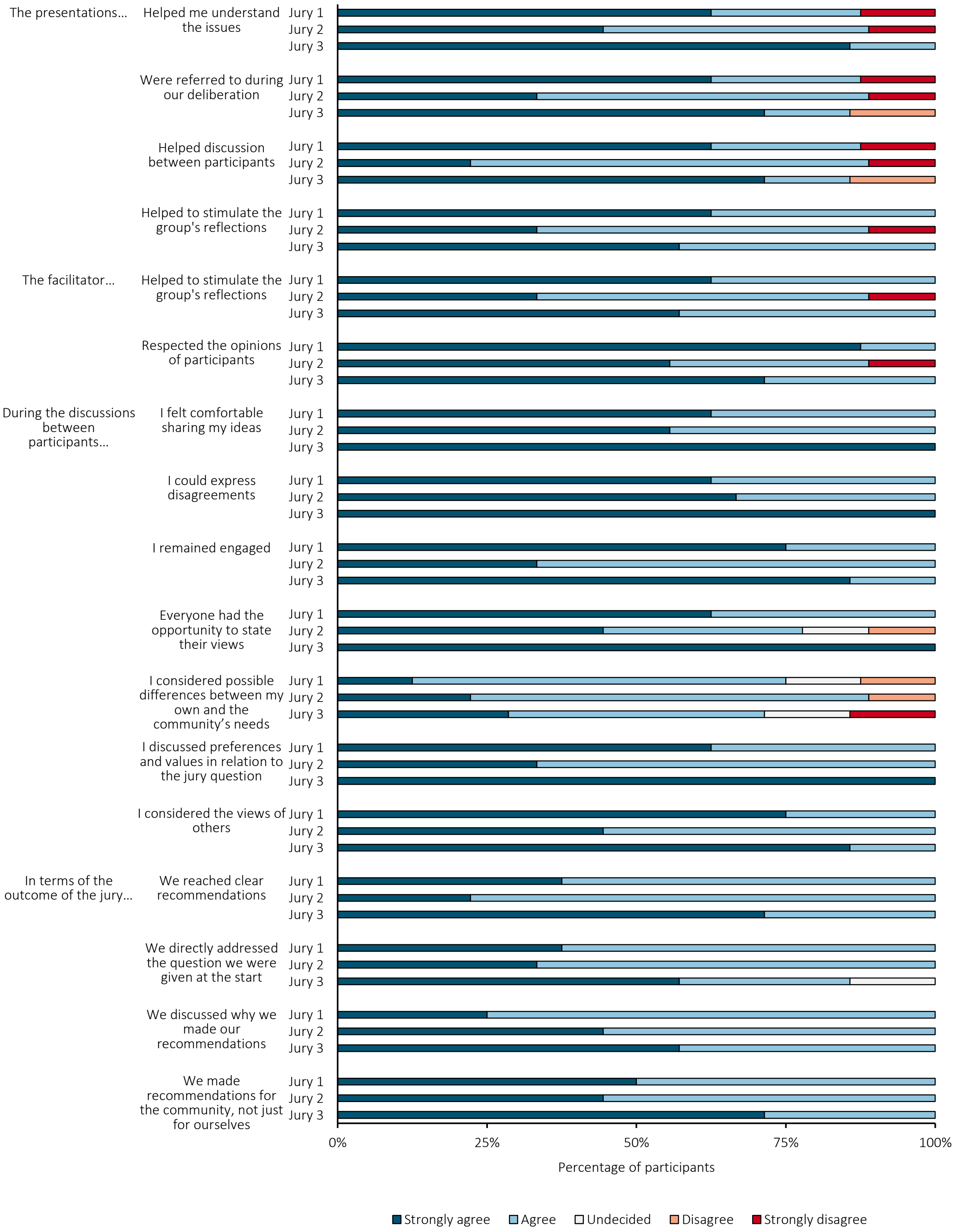

Jury 1 (online) n=8; jury 2 (in-person) n=9; jury 3 (online) n=7 participants.
