# Supplementary material for: Societal views on using risk-based innovations to inform cancer screening and referral policies: findings from three community juries
Source: BMC Public Health. 2025 Feb 27;25:801. doi: 10.1186/s12889-025-21996-x (PMC11869612; doi:10.1186/s12889-025-21996-x)
Supplement: Supplementary file 2 — Additional file 2: Questionnaires and topic guides. Pre-jury questionnaire. Post-jury questionnaire. Facilitated discussion topic guide outline [file 12889_2025_21996_MOESM2_ESM.pdf]

Societal views on using risk-based innovations to inform cancer screening and referral policies: Findings from three community juries

Dennison RA, Clune RJ, Waller J, Tung J, John SD, Moorthie SA, Usher-Smith JA

Additional file 2

Pre-jury questionnaire

Introduction

Thank you for agreeing to complete this questionnaire. Please answer every question. If you are uncertain about how to answer a question, then please select the closest option.

Demographics information

In this first section we would like to ask you a few questions about yourself. These questions allow us to make sure we are including people from a range of different backgrounds and see if different groups of people have different views. We will not be able to identify you from your answers.

How old are you?
- 21–29 years
- 30–39 years
- 40–49 years
- 50–59 years
- 60–69 years
- 70–79 years

What is your sex? *A question about gender identity follows*.
- Male
- Female

Is the gender you identify with the same as your sex registered at birth?
- Yes
- No (please enter your gender identity)

What is your ethnic group? *Choose one option that best describes your ethnic group or background.*
- Asian/Asian British
- Black/African/Caribbean/Black British
- Mixed/Multiple ethnic group
- White
- Other, please describe

What is your highest education level?
- Finished school at or before the age of fifteen
- Completed GCSEs, O Levels or equivalent
- Completed A Levels or equivalent
- Completed further education but not a degree
- Completed a Bachelor's degree
- Completed a Master's degree or PhD
- Other (please specify)

Lifestyle and screening history

The following questions are about your lifestyle and your past screening decisions. Please answer as honestly as you can as this will help us with our analysis.

In general, would you say your health is

- Excellent
- Very Good
- Good
- Fair
- Poor

Have your parents or any brothers or sisters ever had cancer?

- Yes
- No
- Don't know/prefer not to say

Has anyone else close to you (e.g. a partner or close friend) ever had cancer?

- Yes
- No
- Don't know/prefer not to say

Have you ever taken part in any of the following screening programmes? (Please note that they are not offered to everyone). *Information is in the format of description (what it involves, who it is offered to).*

	Yes	No	Not eligible/ haven't been invited
Abdominal aortic aneurysm screening (ultrasound, men aged over 65)			
Bowel cancer screening (poo sample or colonoscopy, men and women aged 60-74)			
Breast cancer screening (mammogram, women aged 50-70)			
Cervical cancer screening (smear test, women aged 25-64)			

Thoughts and beliefs about cancer

The next section asks about your thoughts and beliefs about cancer. Please answer as honestly as you can as this will help us with our analysis. The questions are about your opinion and so there is not a correct answer.

Below are some statements that are sometimes made about cancer. For each of these statements how much do you agree or disagree with them?

These days, many people with cancer can expect to continue with normal activities and responsibilities.

- Strongly agree
- Agree
- Neither agree nor disagree
- Disagree
- Strongly disagree

Most cancer treatment is worse than the cancer itself.

- Strongly agree
- Agree
- Neither agree nor disagree
- Disagree
- Strongly disagree

I would NOT want to know if I had cancer.

- Strongly agree
- Agree
- Neither agree nor disagree
- Disagree
- Strongly disagree

Cancer can often be cured.

- Strongly agree
- Agree
- Neither agree nor disagree
- Disagree
- Strongly disagree

Going to the doctor as quickly as possible after noticing a symptom of cancer could increase the chances of surviving.

- Strongly agree
- Agree
- Neither agree nor disagree
- Disagree
- Strongly disagree

Some people think that a diagnosis of cancer is a death sentence. To what extent do you agree or disagree that a diagnosis of cancer is a death sentence?

- Strongly agree
- Agree
- Neither agree nor disagree
- Disagree
- Strongly disagree

Compared with other people the same age and sex as you, what do you think your chances of getting cancer in the next 10 years are?

- Much below average
- Below average
- Same as average
- Above average
- Much above average

Screening attitudes

Below are some statements about cancer screening. For each of these statements, how much do you agree or disagree with them?

If I feel well, it is not necessary to have cancer screening.

- Strongly agree
- Agree
- Neither disagree nor agree
- Disagree
- Strongly disagree

If I follow a healthy lifestyle such as a balanced diet and regular exercise, I don't feel it is necessary to have regular cancer screening.

- Strongly agree
- Agree
- Neither disagree nor agree
- Disagree
- Strongly disagree

I see a doctor or have cancer screening only when I have a health problem.

- Strongly agree
- Agree
- Neither disagree nor agree
- Disagree
- Strongly disagree

Online privacy attitudes and behaviours

One of the topics we will cover in the juries involves use of personal data and privacy.

In general, how concerned are you about your privacy while you are using the internet?

- Extremely
- Very
- Moderately
- Slightly
- Not at all

Are you concerned that you are asked for too much personal information when you register or make purchases online?

- Extremely
- Very
- Moderately
- Slightly
- Not at all

Are you concerned who might access your medical records electronically?

- Extremely
- Very
- Moderately
- Slightly
- Not at all

Do you watch for ways to control what people send you online (such as check boxes that allow you to opt-in or opt-out of certain offers)?

- Never
- Rarely
- Sometimes
- Often
- Always

Data use for grouping people based on risk

During the community juries, we will discuss your views on calculating people's risk of cancer using data from a variety of different sources. We will also discuss your views on using these risk estimates to make decisions about their access to further healthcare such as tests. At this stage:

Do you think it is a good idea to use existing health data (such as medical records) to understand people's cancer risk?

- Yes, definitely
- Yes, maybe
- I'm not sure
- No, maybe not
- No, definitely not

Do you think it is a good idea to collect and use extra health data (such as genetic tests or tests that can be done at home or in a clinic where the result is immediately available) to understand people's cancer risk?

- Yes, definitely
- Yes, maybe
- I'm not sure
- No, maybe not
- No, definitely not

Do you think it is a good idea to use data that is not currently connected with healthcare (such as shopping habits from loyalty cards or information about people who live in their postcode/region) to understand people's cancer risk?

- Yes, definitely
- Yes, maybe
- I'm not sure
- No, maybe not
- No, definitely not

Do you think it is a good idea to use this information to make decisions about their access to further healthcare?

- Yes, definitely
- Yes, maybe
- I'm not sure
- No, maybe not

End

Post-jury questionnaire

Thank you for taking part in the community juries. For the final part of this study, we invite you to complete the follow-up questionnaire below.

Please answer every question. If you are uncertain about how to answer a question, then please select the closest option.

Data use for grouping people based on risk.

Now that you have taken part in the jury and heard more about this:

Do you think it is a good idea to use existing health data (such as medical records) to understand people's cancer risk?

- Yes, definitely
- Yes, maybe
- I'm not sure
- No, maybe not
- No, definitely not

Do you think it is a good idea to collect and use extra health data (such as genetic tests or tests that can be done at home or in a clinic where the result is immediately available) to understand people's cancer risk?

- Yes, definitely
- Yes, maybe
- I'm not sure
- No, maybe not
- No, definitely not

Do you think it is a good idea to use data that is not currently connected with healthcare (such as shopping habits from loyalty cards or information about people who live in their postcode/region) to understand people's cancer risk?

- Yes, definitely
- Yes, maybe
- I'm not sure
- No, maybe not
- No, definitely not

Do you think it is a good idea to use this information to make decisions about their access to further healthcare?

- Yes, definitely
- Yes, maybe
- I'm not sure
- No, maybe not

Your experience of the community jury

We would also like to know how well you feel the jury went. This will help us to plan future studies as well as whether we should run them online or face-to-face.

Overall, the presentations by the experts…

	Strongly disagree	Disagree	Undecided	Agree	Strongly agree
Helped me understand the issues					
Were referred to during our deliberation					
Helped discussion between community jury members					

Additional comments:

Overall, the facilitator…

	Strongly disagree	Disagree	Undecided	Agree	Strongly agree
Helped to stimulate the group's reflections					
Respected the opinions of participants					

Additional comments:

Overall, during the discussions between jurors…

	Strongly disagree	Disagree	Undecided	Agree	Strongly agree
I felt comfortable sharing my ideas					
I could express disagreements					
I remained engaged throughout the experience					
Everyone was offered the opportunity to state their views					
I considered there might be a difference between what I would want for myself, and what I would recommend for the community as a whole					
I discussed my preferences and values in relation to the jury question					
I considered the views of others during the deliberation					

Additional comments:

The outcome of the jury…

	Strongly disagree	Disagree	Undecided	Agree	Strongly agree
Our community jury reached a clear recommendation/s					
Our community jury's recommendation/s directly addressed the question we were given at the start					
We discussed why we made our recommendations					
We made our recommendations for the community as a whole not just for ourselves.					

Additional comments:

Did you take part in an online or in-person jury?

- o Online
- o In-person (face-to-face)

Please rate your overall experience of the community jury:

Extremely negative – 0 1 2 3 4 5 6 7 8 9 10 – Extremely positive

Please rate how you would anticipate your experience to be if you had taken part [[opposite setting]]:

Extremely negative – 0 1 2 3 4 5 6 7 8 9 10 – Extremely positive

How well do you think the [[setting]] setting helped you to:

	Not at all well	Slightly well	Moderately well	Very well	Extremely well
Participate in and contribute to the jury					
Learn and understand new information					
Feel a sense of community with the other participants					

What did you **like** about taking part [[setting]]?

What did you **not like** about taking part [[setting]]?

Which setting (online, in person or hybrid) would you prefer for future community juries?

- Online
- In-person (face-to-face)
- Hybrid (both online and in-person/face-to-face parts)

How strongly do you prefer the setting you selected above compared to the alternatives?

- No preference
- Prefer slightly
- Prefer a moderate amount
- Prefer a lot

Additional comments:

Other comments (OPTIONAL): Do you have anything to add about your experience on this community jury?

Facilitated discussion topic guide outline

Discussion 1

Opportunity to ask experts outstanding questions (to email).

Thoughts on the principles heard about yesterday of using different data to estimate people's cancer risk. Pros and cons?

Thoughts on the principle of using risk result to change parts of their healthcare. Pros and cons?

Initial thoughts on using [[genetics or polygenic risk / data on where people live / minimally invasive tests / continuous monitoring biomarkers / AI and machine learning / wearable or mobile technology]] to estimate people's risk of cancer.

> And to change parts of their healthcare.
>
> Any situations in which this would/wouldn't be acceptable?

Is there a difference between using this approach in individuals with or without symptoms? Why? Impact of degree/type of symptoms?

Comments on using data that requires action by individuals to collect, that happens automatically, or both. How do you think people in the UK would feel if they were asked to provide some of this data/if the data were collected automatically?

Comments on data that are collected continuously (e.g., wearable device), at single points (e.g., health records), or both. How do you think people in the UK would feel?

Would you prefer to opt-in or opt-out of your data being used in this way? Would it be good for them? Would they have reservations? What would motivate them to allow it? Is there anything that would change their opinion?

Are there any other factors that you think are important to consider in this decision?

Discussion 2

Burden: How much effort or time do you think it is acceptable to ask people to make to provide information to calculate their cancer risk?

Self-efficacy: How confident did you feel that the data we have been talking about could be collected? What do you think should happen if it isn't?

Opportunity costs: Do you think this would be costly (privacy or values)?

Ethicality: To what extent do you think this would make cancer screening and early detection fairer than the current system?

Intervention coherence: How important do you think it is for the public to understand their cancer risk / how their risk is calculated / how this affects screening/diagnostic decisions? What should they be told about it?
